# Compound Heterozygous Mutations in *SLC30A2/ZnT2* Results in Low Milk Zinc Concentrations: A Novel Mechanism for Zinc Deficiency in a Breast-Fed Infant

**DOI:** 10.1371/journal.pone.0064045

**Published:** 2013-05-31

**Authors:** Naoya Itsumura, Yasuji Inamo, Fumiko Okazaki, Fumie Teranishi, Hiroshi Narita, Taiho Kambe, Hiroko Kodama

**Affiliations:** 1 Division of Integrated Life Science, Graduate School of Biostudies, Kyoto University, Kyoto, Japan; 2 Department of Pediatrics and Child Health, Nihon University School of Medicine, Tokyo, Japan; 3 Department of Food Science, Kyoto Women's University, Kyoto, Japan; 4 Department of Pediatrics, Teikyo University School of Medicine, Tokyo, Japan; 5 Department of Health and Dietetics, Faculty of Health and Medical Sciences, Teikyo Heisei University, Tokyo, Japan; University of South Florida College of Medicine, United States of America

## Abstract

Zinc concentrations in breast milk are considerably higher than those of the maternal serum, to meet the infant's requirements for normal growth and development. Thus, effective mechanisms ensuring secretion of large amounts of zinc into the milk operate in mammary epithelial cells during lactation. ZnT2 was recently found to play an essential role in the secretion of zinc into milk. Heterozygous mutations of human ZnT2 (hZnT2), including H54R and G87R, in mothers result in low (>75% reduction) secretion of zinc into the breast milk, and infants fed on the milk develop transient neonatal zinc deficiency. We identified two novel missense mutations in the *SLC30A2/ZnT2* gene in a Japanese mother with low milk zinc concentrations (>90% reduction) whose infant developed severe zinc deficiency; a T to C transition (c.454T>C) at exon 4, which substitutes a tryptophan residue with an arginine residue (W152R), and a C to T transition (c.887C>T) at exon 7, which substitutes a serine residue with a leucine residue (S296L). Biochemical characterization using zinc-sensitive DT40 cells indicated that the W152R mutation abolished the abilities to transport zinc and to form a dimer complex, indicating a loss-of-function mutation. The S296L mutation retained both abilities but was extremely destabilized. The two mutations were found on different alleles, indicating that the genotype of the mother with low milk zinc was compound heterozygous. These results show novel compound heterozygous mutations in the *SLC30A2/ZnT2* gene causing zinc deficiency in a breast-fed infant.

## Introduction

Zinc has a unique and extensive role in numerous biological processes. It is required for structural and catalytic components, and as a signaling factor [1]–[3]. Thus, zinc deficiency can result in growth restriction, immune system dysfunction, skin lesions, alopecia and neurological disorders (reviewed in [4]–[6]). Symptomatic zinc deficiency has been reported in infants. Most reported cases are breast-fed preterm infants [7]–[10], because the zinc concentration in human milk is much lower than that of cow's milk, and the demand for zinc increases rapidly in thriving preterm infants [11]. Zinc deficiency may also occur in breast-fed full-term infants, although it is rare [1]–[17]. Zinc deficiency in breast-fed full-term infants is sometimes caused by congenital *acrodermatitis enteropathica* (OMIM201100), which is caused by a mutation in the *SLC39A4/ZIP4* gene [18]–[21], and results in reduced intestinal zinc absorption [12], [16]. However, it may also be caused by low zinc concentrations in breast milk (OMIM608118) [13]–[15], [17]. The symptoms of zinc deficiency caused by low levels of zinc in breast milk only develop during breast feeding, and do not reoccur after weaning [22], which discriminates this condition from congenital *acrodermatitis enteropathica.*

Pedigree analysis has shown that the condition that predisposes mothers to produce zinc-deficient milk is hereditary [22]. Recent genetic studies have indicated that the condition can be caused by mutations in the *SLC30A2/ZnT2* gene [14], [17]. Thus far, two mutations (in H54R and G87R) have been identified in *SLC30A2/ZnT2.* Both mutations result in milk zinc deficiency in the heterozygous condition, which suggests haploinsufficiency or dominant negative mechanisms [14], [17]. In mice, homozygous mutations in the *SLC30a4/Znt4* gene result in impaired secretion of zinc into the milk [23]. This causes the “lethal milk” phenotype (OMIM602095), a term derived from the fact that pups nursed by affected dams die before weaning [23].

In this study we identified two novel missense mutations in the *SLC30A2/ZnT2* gene in a Japanese mother who secreted zinc-deficient breast milk, causing her breast-fed infant to develop severe zinc deficiency that was reversed by zinc replacement therapy. Using DT40 cells, in which we have previously shown the biochemical characteristics of a number of zinc transporters including ZnT and ZIP [24]–[29], we characterized one of these missense mutations at the molecular level as a loss-of-function mutation, while the other retained its functions but was markedly destabilized. The two missense mutations were located on different alleles, indicating that the low milk zinc is caused by compound heterozygous mutations of *SLC30A2/ZnT2* gene. These results show a novel molecular mechanism underlying zinc deficiency in a breast-fed infant. We also discuss the effects of both mutants and two previously identified H54R and G87R mutants on breast milk zinc levels from the perspective of their zinc transport activity and protein stability as evaluated using our system using DT40 cells.

## Materials and Methods

### Clinical data

**Figure 1:**
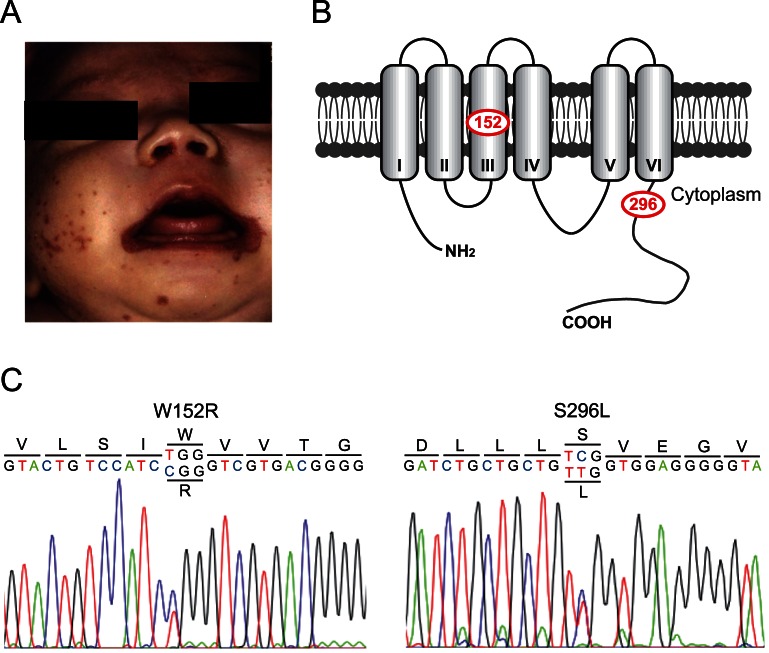
Identification of two missense mutations in the *SLC30A2/ZnT2* gene in the mother of a zinc-deficient infant. (**A**) Photograph of an affected infant with severe zinc deficiency. The dermatitis was erythematous and erosive, particularly around the infant's mouth. (**B**) Predicted topology of hZnT2 indicating the positions of the W152R and S296L substitutions found in this study. (**C**) Electropherograms showing SLC30A2/*ZnT2* mutations in the affected mother. W152R and S296L mutations were found at exons 4 and 7 on different alleles.

The patient was a full-term male baby (gestational age 37 weeks; birth weight 2,518 g) who had been fully fed on breast milk from his mother. Dermatitis had been found since a postnatal age of 13 days. The dermatitis was erythematous and erosive, particularly around his mouth, diaper region and fingers (Figure 1A and Figure S1). The dermatitis could not be improved by topical anti-inflammatory drugs, including corticosteroids. The patient had persistent diarrhea and alopecia, and his weight gain was poor (10 g/day). A diagnosis of zinc deficiency was established at a postnatal age of 4 weeks by the attending pediatrician (Y. I.) based on the clinical presentation, and was confirmed by low serum zinc levels (11 μg/dL; normal level 63–81 μg/dL). The mother's breast milk and serum zinc levels were subsequently evaluated. The breast milk zinc level (0.02 mg/dL) was lower than the normal level expected during the fourth week of lactation (0.2 mg/dL) [30], [31]. However, her serum zinc level was normal (92 μg/dL). The infant was given oral zinc replacement therapy with Polaprezinc (JAN/INN: (C_9_H_12_N_4_O_3_Zn)n; 3 mg/kg/day) and continued breast feeding. The skin lesions began to improve within 28 days after the initiation of therapy and were completely cured after 6 months of therapy. Zinc supplementation was stopped after the start of the weaning diet, after which the infant's serum zinc level remained within normal ranges. The dermatitis never reoccurred. The patient's physical and mental development is now normal.

This study was approved by the institutional review board of the Nihon University Nerima-Hikarigaoka Hospital, Nihon University School of Medicine, by the institutional review board of Teikyo University School of Medicine (No. 09–066), and by the Ethics Committee of Kyoto University Graduate School and Faculty of Medicine. Written consent was obtained from the patient's mother at Nihon University Nerima-Hikarigaoka Hospital. The medical history of the family was established by an interview with the mother. Serum and milk zinc concentrations were determined by independent laboratory testing and obtained from the patient's medical records.

### Sequencing of SLC30A2/ZnT2 and SLC30A4/ZnT4

Genomic DNA was isolated from whole blood of the mother using a commercial genomic DNA extraction kit (Genomix; Talent SRL, Trieste, Italy). All exons containing coding regions (including splicing sites) of *SLC30A2/ZnT2* and *SLC30A4/ZnT4*, the sequence in and around the promoter region and the sequences of potential STAT5 binding sites functional as the response element for prolactin in *SLC30A2/ZnT2* gene were amplified by PCR using KOD Plus (TOYOBO, Osaka, Japan). The amplified fragments were directly sequenced in both directions using the same primers. PCR primer information is provided in Tables S1 and S2. To confirm each mutation on the allele, PCR products amplified between exons 4 and 7 were subcloned into TOPO TA Vector pCR II (Invitrogen, Carlsbad, CA) and sequenced with appropriate primers. To perform sequence analysis of the *SLC30A2/ZnT2* promoter region, the following primers were used: hZnT2-MRE-Fw: 5'-AGC AGA GAG GCA CTC AGT GAG GAC CCA AGC-3'; hZnT2-MRE-Rv: 5'-AGC CGC CCC GCC GAG TGC GCC CTG AAA GTT-3'. The following sequences are used for the alignment of the promoter regions of *ZnT2* genes: Accession Nos. are genomic sequences for humans; NT_004610.19, Rat; NW_047725.1 and Mouse; NT_187033.1.

### Cell culture and transfection

Chicken B lymphocyte-derived DT40 cells [32] were maintained as described previously [28]. DT40 cells deficient in *ZnT1*, *metallothionein* (*MT*) and *ZnT4* genes (*ZnT1* ^−/−^ *MT* ^−/−^ *ZnT4* ^−/−^ cells) were established as described elsewhere (manuscript in preparation). To monitor the stability of the hZnT2 protein, cycloheximide (CHX, Sigma, St. Louis, MO) was added to the culture medium at a final concentration of 50 μg/ml. Proteasome inhibitor MG132 (Peptide Institute Inc., Osaka, Japan) or lysosome inhibitor bafilomycin A1 (Sigma) was added into the medium 2 h before treatment with CHX at final concentrations of 30 μM or 30 nM, respectively. Cells were removed and washed once with phosphate-buffered saline after 0, 1, 2, 4 or 8 h of incubation. To evaluate cell viability against extracellular high zinc, the cells were cultured in the presence of 50–90 μM ZnSO_4_ for 72 h. The numbers of viable cells, judged by exclusion of trypan blue, were then counted and relative viability was determined. DNA transfection into DT40 cells was carried out by electroporation as previously described [28].

### Plasmid construction

Plasmids to express wild-type (WT) or mutant carboxyl terminally HA- or FLAG-tagged human ZnT2 (hZnT2-HA or hZnT2-FLAG) were constructed by inserting each cDNA into pA-Neo or pA-Puro vectors [29]. Introduction of mutation into *hZnT2* cDNA was carried out by two-step PCR methods, and amplified cDNAs were sequenced in both directions. All plasmids were linearized with appropriate restriction enzymes prior to electroporation. The MT-luciferase reporter plasmid was kindly gifted by Dr. Tomoki Kimura.

### Generation of anti-ZnT2 monoclonal antibody

As an antigen for the ZnT2 antibody, we used the fused proteins consisting of the cytosolic carboxyl terminal portion of hZnT2 (109 amino acid residues from the glycine at amino acid 264 to stop) and maltose binding protein or glutathione S-transferase protein. The hybridoma that produces the anti-hZnT2 antibody was produced as described previously [33]. Ascites was generated by injection of 1×10^7^ hybridoma cells into pristine-primed mice.

### Immunoblotting and immunoprecipitation

Immunoblotting and immunoprecipitation were performed as described previously [29]. The blotted membrane was blocked with blocking solution (5% skim milk and 0.1% Tween-20 in phosphate-buffered saline) and then incubated with monoclonal anti-HA HA-11 (1∶4000 dilution; COVANCE, Emeryville, CA), monoclonal anti-FLAG M2 (1∶4000; Sigma), polyclonal anti-HA (1∶4000; MBL, Nagoya, Japan), polyclonal anti-FLAG (anti-DDDDK; 1∶4000; MBL), or anti-tubulin (1∶20000; Sigma) antibodies in blocking solution. For immunoprecipitation, the membrane factions prepared from cells lysed in NP-40 buffer [25] were rotated with anti-FLAG M2 (1∶200 dilution) or anti-HA HA-11 (1∶200 dilution) antibodies for 1 h prior to the addition of 10 µl of Protein G-Sepharose beads (GE Healthcare, Waukesha, WI). After incubating for 2 h, immunoprecipitates were subjected to immunoblotting as described previously [25]. For the examination of phosphorylation of the hZnT2 proteins, total cellular lysates were prepared from the cells expressing WT or mutant hZnT2 in the presence of phosphatase inhibitor cocktail (SIGMA). Lysates were resolved by Phos-tag SDS-PAGE (Wako Pure Chemical, Osaka, Japan), followed by standard immunoblotting. Horseradish peroxidase-conjugated anti-mouse or anti-rabbit secondary antibodies (GE Healthcare) were used at a 1∶4000 dilution for detection. Immobilon Western Chemiluminescent HRP Substrates (Millipore, Billerica, MA), or Chemi-Lumi One L (Nacalai Tesque) was used for detection. The fluoroimage was obtained using a LAS1000 plus image analyzer (Fujifilm, Tokyo, Japan). Densitometry quantification of hZnT2 protein was performed using Image Gauge (Fujifilm).

### Luciferase assay

The activities of firefly and renilla luciferase were measured using a dual-luciferase reporter assay system (Promega, Madison, WI) with Lumat LB9501 (Berthold Technologies, Bad Wildbad, Germany) as described previously [26]. After transfection, the cells were pre-cultured for 4 h in fresh medium and then cultured for 24 h in the presence of 25 or 50 μM ZnSO_4_. Firefly luciferase activity was divided by renilla luciferase activity for normalization of transfection efficiency.

### Statistical analyses

All data are depicted as mean ± SD. Statistical significance was determined by Student's t test and accepted at p<0.05.

## Results

### Identification of novel missense mutations in the SLC30A2/ZnT2 gene

**Figure 2:**
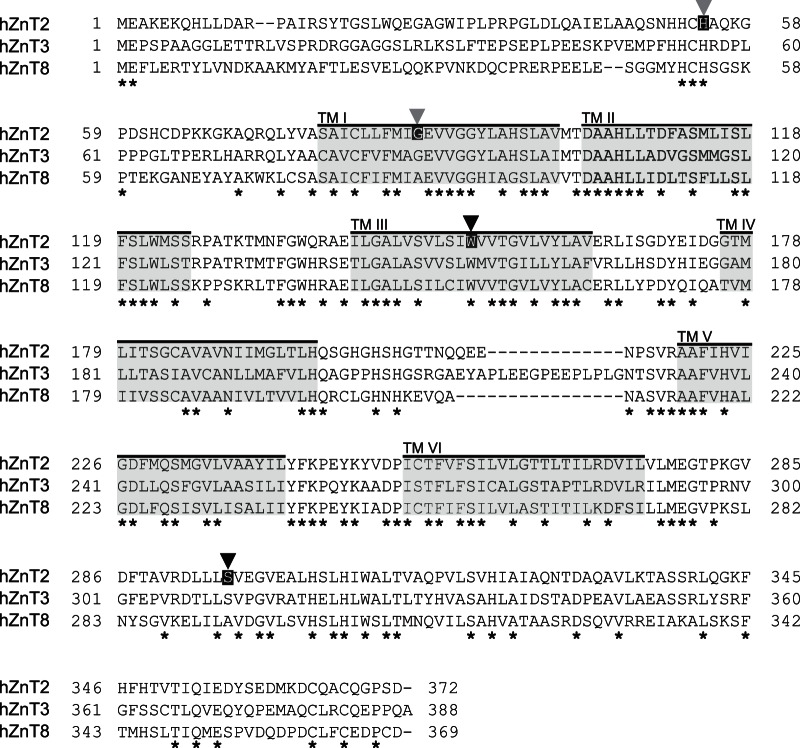
Sequence alignment among hZnT2, hZnT3 and hZnT8. The positions of tryptophan (corresponding to W152 in hZnT2) and serine (S296) residues (indicated by black arrowheads) identified in the affected mother with low milk zinc are completely conserved in hZnT3 and semi-conserved in hZnT8. The positions of histidine (corresponding to H54 in hZnT2) and glycine (G87) residues that have been identified are also indicated by gray arrowheads. Identical amino acids are indicated by *. The putative transmembrane regions, which are predicted by SOSUI (http://bp.nuap.nagoya-u.ac.jp/sosui/) using hZnT2 sequence, are shaded in gray.

**Figure 3:**
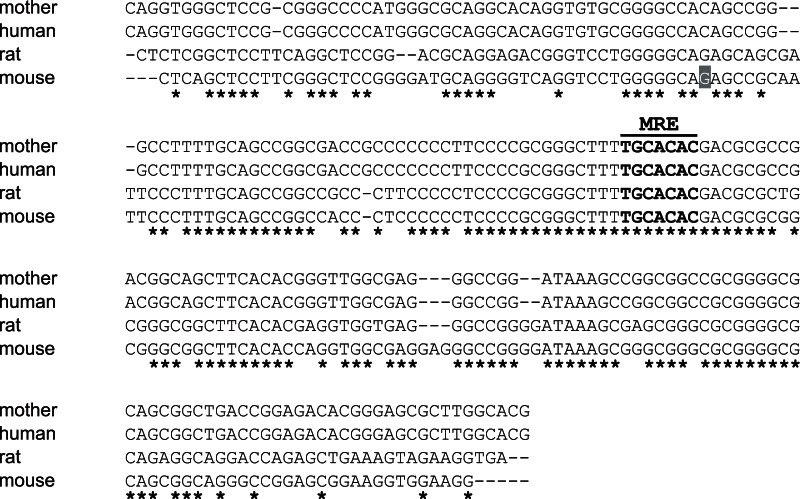
No mutations were found in and around the promoter region of the *SLC30A2/ZnT2* gene. Alignment of the sequences of the mother with low milk zinc (“mother”) and the human genomic sequence deposited in the GenBank database (“human”). To show high homology in this region among mammals, where the MRE is completely conserved, the sequences are also aligned with those of rats and mice deposited in the GenBank database (corresponding regions from −110 to +22 of mouse *Znt2* are shown. The transcription start site is indicated by gray shading). Identical nucleotides are indicated by * and the MRE sequence is indicated with bold letters.

We analyzed all exons and their flanking regions, including splicing sites of *SLC30A2/ZnT2* and *SLC30A4/ZnT4*, using amplified fragments from genomic DNA extracted from the blood of the mother. We found no mutations in the *SLC30A4/ZnT4* gene in the open reading frame or the region predicted to affect protein expression, which is consistent with previous studies [14], [34]. However, two novel missense mutations were found in the *SLC30A2/ZnT2* gene (Figure 1B and C); c.454T>C at exon 4 (referring to the adenine of the start codon in the longer isoform (NP_001004434) as +1), which introduces an arginine residue in place of a tryptophan residue (W152R), and c.887C>T at exon 7, which introduces a leucine residue in place of a serine residue (S296L) (Figure 1B and C). The tryptophan and serine residues are completely conserved in hZnT3 and semi-conserved in hZnT8 (Figure 2). Neither is present in the NCBI single nucleotide polymorphisms (SNPs) (http://www.ncbi.nlm.nih.gov/snp) or expressed sequence tag databases. We also analyzed the sequence in and around the *SLC30A2/ZnT2* promoter region, including the metal response element (MRE) sequence (up to ∼−1.0 kb from the putative transcription start site) [35] (Figure 3), and the sequences of four potential STAT5 binding sites in the −3.3 to −1.9 kb upstream sequence of *SLC30A2/ZnT2*, because the regulation of expression via the MRE and STAT5 binding sites is important for *ZnT2* transcription, in response to zinc [35], [36] or to the lactogenic hormone, prolactin, which is important for zinc uptake in mammary cells and for zinc secretion into the milk [37], [38]. However, no mutations were found in these regions (Figure 3 and data not shown).

The two novel missense mutations were found in different subclones containing PCR-amplified fragments spanning exons 4 through 7 (data not shown), revealing that both were located on separate alleles. These results suggest that either or both mutations caused zinc secretion into the breast milk to be impaired, resulting in severe zinc deficiency in the infant.

### Examination of zinc transport activity of hZnT2 mutants

**Figure 4:**
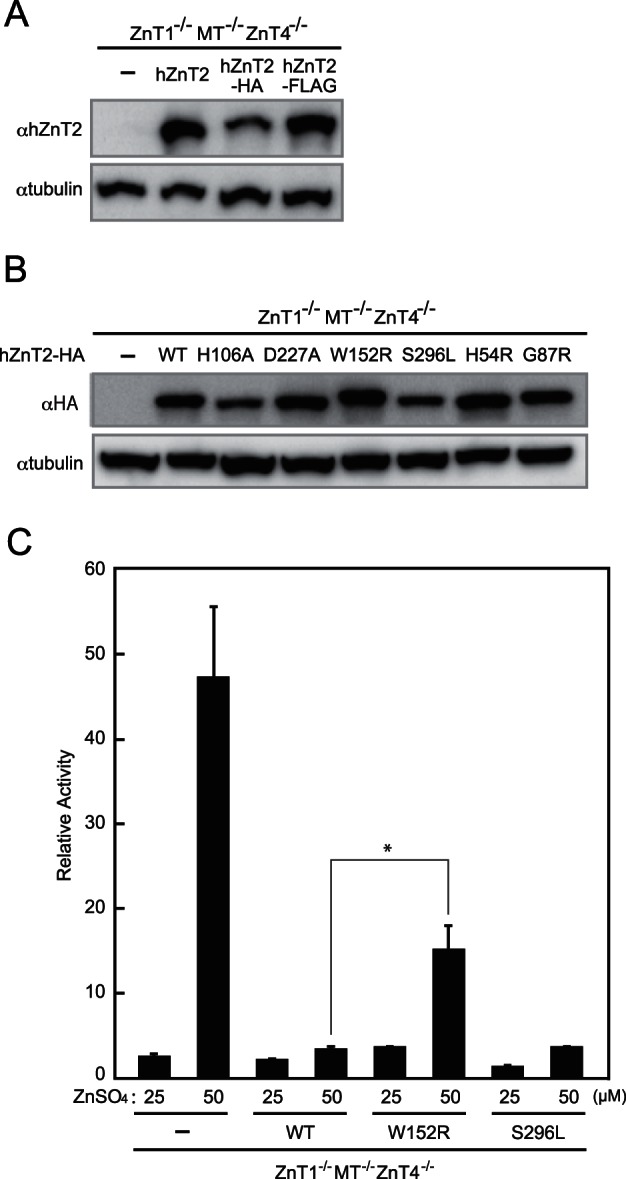
W152R hZnT2 loses the ability to transport zinc, but the S296L hZnT2 does not. **(A)** The carboxyl-terminal epitope tags do not interfere with hZnT2 expression. Untagged and HA- or FLAG-tagged hZnT2 were stably expressed in *ZnT1* ^−/−^ *MT* ^−/−^ *ZnT4* ^−/−^ cells. Immunoblotting was performed using anti-hZnT2 antibody. (**B**) Confirmation of stable expression of the WT hZnT2-HA, W152R, S296L and other mutants of hZnT2-HA in *ZnT1* ^−/−^ *MT* ^−/−^ *ZnT4* ^−/−^ cells. Immunoblotting was performed using an anti-HA antibody. In both (**A**) and (**B**), 20 µg of total cellular protein was loaded onto each lane, and the same membrane was used for detection of both hZnT2 and tubulin. Tubulin is shown as a loading control. (**C**) Effects of zinc on MT-luciferase reporter gene expression in *ZnT1* ^−/−^ *MT* ^−/−^ *ZnT4* ^−/−^ cells stably expressing WT hZnT2-HA, W152R or S296L mutant hZnT2-HA. Relative activity of Luc is shown (the luciferase activity of *ZnT1* ^−/−^ *MT* ^−/−^ *ZnT4* ^−/−^ cells cultured without ZnSO_4_ is defined as 1). Each value is the mean ± SD of triplicate experiments. * denotes a significant difference of relative activity of Luc between the cells expressing WT and W152R mutant hZnT2 (P<0.05).

To investigate if either the W152R mutation, the S296L mutation or both was the cause of the milk zinc deficiency, we examined the zinc transport activities of W152R and S296L hZnT2 mutants. Previous studies have shown that the zinc transport activity of ZnT2 can be detected in cells lacking endogenous ZnT1 and MTs [39]–[41]. We established DT40 cells that show similar zinc-sensitivity by disrupting the *ZnT1, MT* and *ZnT4* genes (*ZnT1* ^−/−^ *MT* ^−/−^ *ZnT4* ^−/−^ cells). *ZnT1* ^−/−^ *MT* ^−/−^ *ZnT4* ^−/−^ cells were sensitive to extracellular high zinc and did not grow in the presence of 60 µM or more ZnSO_4_ (Table 1), which is a very useful phenotype for detecting the zinc transport activity of ZnT transporters, including ZnT2 (data not shown). We expressed the hZnT2 proteins of WT, W152R, S296L and other mutants in *ZnT1* ^−/−^ *MT* ^−/−^ *ZnT4* ^−/−^ cells, and compared their zinc transport activities. Since the carboxyl-terminally fused HA- or FLAG-tags did not interfere with zinc transport activity of hZnT2 (Figure 4A and Table 1), we used the tagged hZnT2 in subsequent studies (Figure 4B). Stable expression of WT hZnT2 clearly reversed the zinc-sensitive phenotype of *ZnT1* ^−/−^ *MT* ^−/−^ *ZnT4* ^−/−^ cells, but expression of the H106A and D227A hZnT2 mutants, in which the essential amino acids for zinc-binding in transmembrane domains are mutated [42], [43], failed to do so (Table 1). This indicates that zinc transport activity of hZnT2 can be evaluated using *ZnT1* ^−/−^ *MT* ^−/−^ *ZnT4* ^−/−^ cells. Stable expression of the W152R hZnT2 mutant failed to reverse the zinc-sensitive phenotype of *ZnT1* ^−/−^ *MT* ^−/−^ *ZnT4* ^−/−^ cells (Table 1), while expression of the S296L hZnT2 mutant did reverse the phenotype, although the effects were slightly less pronounced than those of WT hZnT2 (Table 1). To compare the zinc transport properties of the W152R and S296L mutants with those of H54R and G87R mutants, which were previously identified as mutations causing low milk zinc in the heterozygous condition, we also expressed the latter two mutants in *ZnT1* ^−/−^ *MT* ^−/−^ *ZnT4* ^−/−^ cells. Stable expression of the H54R hZnT2 mutant only moderately reversed the phenotype, and expression of G87R did not affect zinc sensitivity at all (Table 1). These results indicate that a mutation resulting in W152R can be a cause of low levels of zinc in breast milk.

**Table 1:** Evaluation of zinc transport activities of hZnT2 proteins using *ZnT1* ^−/−^ *MT* ^−/−^ *ZnT4* ^−/−^ cells.

			ZnSO_4_ (µM)			
Cells	Expressed gene	50	60	70	80	90
WT	−	+++	+++	+++	+++	+++
*ZnT1* ^−/−^ *MT* ^−/−^ *ZnT4* ^−/−^	−	+++	−	−	−	−
*ZnT1* ^−/−^ *MT* ^−/−^ *ZnT4* ^−/−^	hZnT2	+++	+++	+++	+++	+++
*ZnT1* ^−/−^ *MT* ^−/−^ *ZnT4* ^−/−^	hZnT2-HA	+++	+++	+++	+++	+++
*ZnT1* ^−/−^ *MT* ^−/−^ *ZnT4* ^−/−^	hZnT2-FLAG	+++	+++	+++	+++	+++
*ZnT1* ^−/−^ *MT* ^−/−^ *ZnT4* ^−/−^	H106A hZnT2	+++	−	−	−	−
*ZnT1* ^−/−^ *MT* ^−/−^ *ZnT4* ^−/−^	D227A hZnT2	+++	−	−	−	−
*ZnT1* ^−/−^ *MT* ^−/−^ *ZnT4* ^−/−^	W152R hZnT2	+++	−	−	−	−
*ZnT1* ^−/−^ *MT* ^−/−^ *ZnT4* ^−/−^	S296L hZnT2	+++	+++	+++	++	+
*ZnT1* ^−/−^ *MT* ^−/−^ *ZnT4* ^−/−^	H54R hZnT2	+++	++	+	−	−
*ZnT1* ^−/−^ *MT* ^−/−^ *ZnT4* ^−/−^	G87R hZnT2	+++	−	−	−	−

Viability of the cells exposed to the indicated concentrations of ZnSO_4_ for 72 h was determined by counting the number of viable cells. Relative values presented are evaluations of the averages of three independent experiments. All of hZnT2 mutants were HA-tagged. +++: growing to confluence; ++, +: less growth (20–50% or up to 20% relative to +++); −: not growing.

The results of the restoration experiments regarding the W152R and S296L hZnT2 mutants were confirmed by MT-luciferase reporter assays. When cultured under high-zinc conditions, high luciferase activity was observed in *ZnT1* ^−/−^ *MT* ^−/−^ *ZnT4* ^−/−^ cells and the cells expressing the W152R hZnT2 mutant, but the activity was markedly reduced in the cells expressing the WT hZnT2 or S296L hZnT2 mutant (Figure 4C). These effects can be attributed to a decrease in the cytosolic zinc contents by expression of active hZnT2 with the ability to mobilize zinc into the vesicles where hZnT2 is localized. These results indicate that the W152R hZnT2 mutant is a loss-of-function mutant but the S296L hZnT2 mutant retains the activity.

### Characterization of W152R and S296L hZnT2 mutants as ZnT transporter

**Figure 5:**
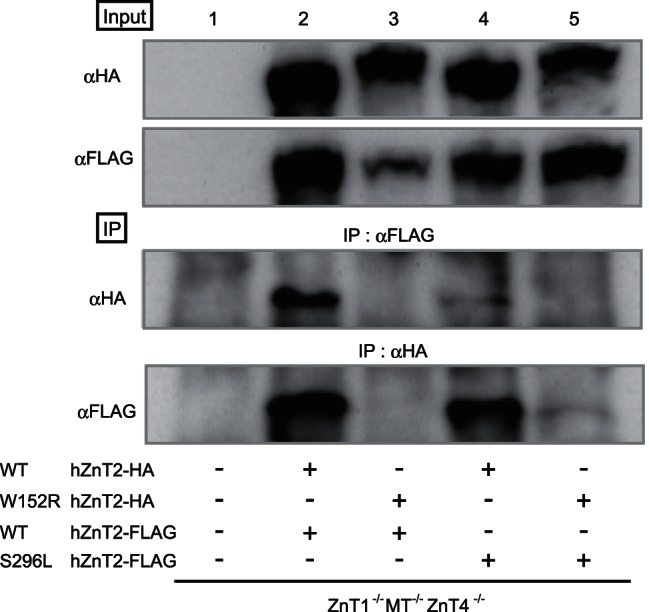
W152R hZnT2 mutant is not dominant negative because it fails to form functional dimers. Tagged-hZnT2 WT or mutants were immunoprecipitated *(IP*) with antibodies against either the FLAG or HA epitopes. The immunoprecipitates were analyzed by immunoblotting using antibodies against the FLAG or HA tags. To estimate the amount of tagged hZnT2 WT and mutant proteins, 10% of each aliquot was subjected to immunoblot analysis (*input* panels). The IP experiments were performed four times, which gave the same results. The panels show the representative results.

Most ZnT transporters, except for ZnT5 and ZnT6, form homo-oligomers (homo-dimers) to mobilize zinc across the cell membrane [17], [25], [28], [42], [44]–[46]. To investigate the ability of each hZnT2 mutant to form functional complex, we established a series of *ZnT1* ^−/−^ *MT* ^−/−^ *ZnT4* ^−/−^ cells expressing various combinations of WT or mutant hZnT2 whose carboxyl-terminal ends were tagged with HA or FLAG epitopes, and performed co-immunoprecipitation experiments. We first confirmed that a functional interaction occurs between WT hZnT2-HA and WT hZnT2-FLAG (Figure 5, lane 2), verifying that this strategy works well, like in the cases of ZnT5 and ZnT6 or ZnT7 [25], [28]. The interaction between WT hZnT2-HA and S296L hZnT2-FLAG was confirmed (Figure 5, lane 4), consistent with preservation of the zinc transport activity of the S296L mutant. In contrast, almost no interaction was found between WT hZnT2-FLAG and W152R hZnT2-HA (Figure 5, lane 3), and between W152R hZnT2-HA and S296L hZnT2-FLAG (Figure 5, lane 5), although a very faint band could be detected. These results exclude the possibility that the W152R mutant has dominant negative effects on the zinc transport activity of WT hZnT2, like the G87R mutant recently identified [17], and concurrently suggest that the S296L mutant must have some defects directly resulting in a decrease in zinc secretion into breast milk.

### S296L mutation causes hZnT2 protein destabilization

Topology prediction programs such as SOSUI (http://bp.nuap.nagoya-u.ac.jp/sosui/) and HMMTOP (http://www.enzim.hu/hmmtop) predict that the S296L mutation changes the topology of hZnT2 by creating an additional transmembrane domain in the carboxyl terminal region (data not shown), which first led us to speculate that the S296L hZnT2 mutant may be a loss-of-function mutation. However, our results excluded this possibility because the S296L hZnT2 mutant still retained the abilities to transport zinc and form the dimer complex. Zinc transport activity of the S296L mutant appeared to be slightly reduced (Table 1), but that was unlikely to be a predominant reason for the low zinc content of the breast milk.

**Figure 6:**
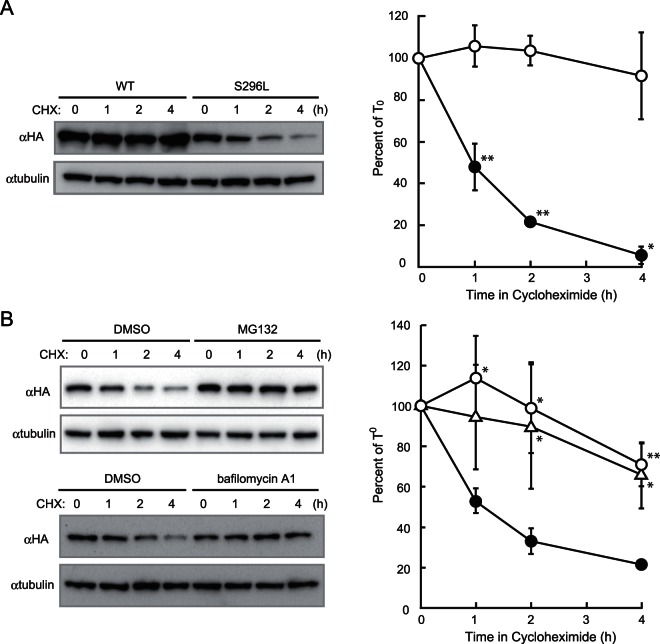
S296L mutation causes hZnT2 destabilized. (**A**) The expression level of the hZnT2 protein at each time point. The *ZnT1* ^−/−^ *MT* ^−/−^ *ZnT4* ^−/−^ cells expressing WT hZnT2 or S296L mutant were treated with CHX and collected periodically over 4 h. Immunoblot analysis was performed to monitor hZnT2 levels (*left* panel). The band intensities of hZnT2 protein (○, WT; •, S296L mutant) are shown as the percentage of the intensity at 0 h (T_0_) after normalized by that of tubulin at each time (*right* panel). * and ** denote a significant difference between expression levels of the WT and S296L mutant hZnT2 at each time point (* P<0.05, ** P<0.01) (**B**) Lysosome inhibitor bafilomycin A1 and proteasome inhibitor MG132 block the degradation of S296L hZnT2 mutant. Immunoblot analysis (*left* panel) and the band intensities of hZnT2 protein (○, MG132; Δ, bafilomycin A1; • no inhibitor, *right* panel) are shown. In the right panels of both (**A**) and (**B**), each value is the mean ± SD of triplicate experiments. The same membrane was used for detection of both hZnT2 and tubulin. Tubulin is shown as a loading control. * and ** denote significant differences between expression levels in the absence and presence of bafilomycin A1 or MG132 at each time point (* P<0.05, ** P<0.01).

Since we evaluated the functions of the S296L mutant by an overexpression system using a strong promoter (β-actin), which may mask the regulation of the expression of the S296L mutant protein, we more closely examined the protein stability of the S296L hZnT2 mutant. To perform this experiment, *ZnT1* ^−/−^ *MT* ^−/−^ *ZnT4* ^−/−^ cells expressing the WT hZnT2 or S296L hZnT2 mutants were treated with CHX to block further protein synthesis, and the protein expression levels were monitored periodically over 4 h by immunoblotting. No significant differences were observed in WT hZnT2 expression, but marked decreases were found in the S296L hZnT2 mutant (Figure 6A). The decrease was blocked by the proteasome inhibitor MG132 and bafilomycin A1, an inhibitor of the vacuolar type H^+^-ATPase that inhibits the lysosomal pathway of protein degradation (Figure 6B). Thus, the S296L hZnT2 mutant is degraded via intracellular protein degradation systems, including the ubiquitin-proteasome and the lysosome pathways. The protein levels of the S296L hZnT2 mutant at 4 h were less than 20% of the levels at 0 h, indicating that the mutant is markedly destabilized. Reduced protein expression levels by mutations have been shown to result in decreased zinc transport activity in Zip4 [18]. Thus, this protein instability of the S296L hZnT2 mutant is a likely reason why the mutation causes decreased zinc transport into breast milk. Since the two missense mutations of the *SLC30A2/ZnT2* gene causing W152R and S296L substitutions were found on different alleles, we conclude that the compound heterozygous mutations in *SLC30A2/ZnT2* are responsible for the low zinc levels in the breast milk of the Japanese mother in our study, which caused severe zinc deficiency in her infant.

### Protein stability of W152R, H54R and G87R hZnT2 mutants

**Figure 7:**
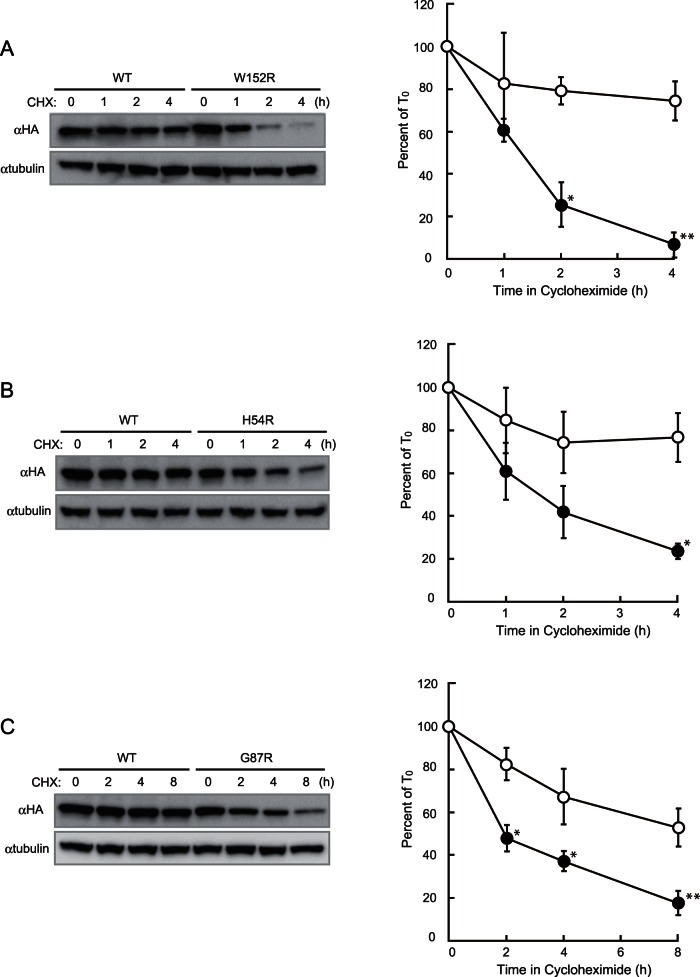
Protein stability of W152R, H54R and G87R hZnT2 mutants. The expression levels of the WT (○) and W152R (•) mutant hZnT2 proteins (**A**), the WT (○) and H54R (•) mutant hZnT2 proteins (**B**), and the WT (○) and G87R (•) mutant hZnT2 proteins (**C**) at each time point. Immunoblot analysis was performed to monitor hZnT2 levels (*left* panel), as described in Figure 6. In the right panels of (**A**) – (**C**), each value is the mean ± SD of triplicate experiments. The same membrane was used for detection of both hZnT2 and tubulin. Tubulin is shown as a loading control. * and ** denote a significant difference between expression levels of the WT and W152R, H54R or G87R mutant hZnT2 at each time point (* P<0.05, ** P<0.01).

Last, we examined the protein stability of the W152R, H54R and G87R hZnT2 mutants in *ZnT1* ^−/−^ *MT* ^−/−^ *ZnT4* ^−/−^ cells. The protein expression levels of each mutant were monitored periodically by immunoblotting in the same manner as in Figure 6. A marked decrease was found in the expression level of the W152R hZnT2 mutant (Figure 7A), and a moderate decrease was found in the expression of the H54R mutant (Figure 7B), although its instability was less profound than that of the S296L mutant. The G87R mutant was previously suggested to have reduced stability [17], but we only detected a significant decrease at 8 h (Figure 7C). Thus, the G87R hZnT2 mutant, which completely lacks any zinc transport activity (Table 1), is destabilized, but the instability is much less than those of the other three mutants, including S296L.

## Discussion

We report two novel missense mutations in the *SLC30A2/ZnT2* gene causing W152R and S296L substitutions in a mother who produced zinc-deficient breast milk (>90% reduction), resulting in severe zinc deficiency in her breast-fed infant. Results from our biochemical experiments indicate that the mother is compound heterozygous for these two mutations. This conclusion was based on the following: First, the W152R hZnT2 mutant is a loss-of-function mutation resulting in complete failure of zinc transport. It does not have dominant negative effects because it also failed to form a functional dimer complex. Second, the S296L hZnT2 mutant was markedly destabilized in the cells compared with WT hZnT2, and more prominent than the H54R and G87R mutants of hZnT2. However, overexpression of this mutant preserved the abilities to transport zinc and form a functional dimer complex. Third, the two mutations were found on different alleles. Our conclusion contrasts with those of two previous reports regarding H54R and G87R mutants, which caused reductions (>75%) in the zinc content of breast milk and thus caused neonatal zinc deficiency in the heterozygous condition. In these studies the H54R mutation resulted in reduced zinc secretion because of aggresomal accumulation of hZnT2 [14], and the G87R hZnT2 mutation had dominant negative effects [17]. Given the fact that heterozygous mutations result in low secretion of zinc into the breast milk, the possibility that the S296L hZnT2 mutant may dominant-negatively impair the functions of WT hZnT2 by forming a dimer complex with it and resulting in its degradation should be closely considered. We cannot completely exclude this possibility, but speculate that the dominant negative effects of the S296L hZnT2 mutant would be much less pronounced, if present at all, because its ability to form a dimer complex appears to be weaker than that of WT hZnT2 (Figure 5). More studies are required to verify this hypothesis to help prevent zinc deficiency in breast-fed infants.

As described above, the zinc levels in the breast milk of the Japanese mother with compound heterozygous mutations were reduced by >90%, which was more severe than the decrease in breast milk zinc levels previously reported in mothers with heterozygous mutations of H54R or G87R (>75% reduction). These differences are likely to be related to the timing of the appearance of dermatitis in the affected infants. Dermatitis appears at 3 to 6 months after birth in the case of H54R mutation, or at 2 to 2.2 months after birth in the case of G87R mutation. In contrast, the dermatitis appeared in the affected infant in the present study on day 13 after birth, which is a more rapid onset and likely due to the lower breast milk zinc concentrations following birth and a faster depreciation of the infant's zinc stores than found in the other studies. The more severe decrease in the breast milk zinc levels in the Japanese mother in our study may be explained by compound heterozygosity for S296L and W152R mutations, which caused markedly destabilized and complete loss-of-function in the hZnT2 protein, respectively, and therefore resulted in a significantly greater decrease in the net zinc transport by hZnT2 in the mammary cells when compared with the zinc transport in the heterozygous condition of H54R or G87R.

The S296L hZnT2 mutant was markedly destabilized. The serine residue at amino acid 296 is predicted to be a phosphorylation site by prediction programs such as NetPhos (http://www.cbs.dtu.dk/services/NetPhos/) or PhosphoMotif Finder (http://www.hprd.org/PhosphoMotif_finder). This suggests that stability of the hZnT2 protein may be regulated by its phosphorylation, as has been found for a number of proteins, including PTEN [47]. We examined this possibility by several biochemical experiments using Phos-tag SDS-PAGE, which can resolve phosphorylated proteins by SDS-PAGE [48], but our results did not support the hypothesis (data not shown). The substitution of the serine residue at amino acid 296 to leucine may change the substructure of the cytosolic carboxyl terminal region of hZnT2 and affect the stability of the protein, because this region is shown to be important for the regulation of protein-protein interactions in various ZnT transporters [49].

The W152R hZnT2 mutant is a loss-of-function mutant that neither transports zinc nor forms a functional dimer complex. Although the reasons are as yet unclear, we can give some insights based on the X-ray structure of the ZnT homologue YiiP of *E. coli* and information from extensive search of amino acid residues affecting zinc transport activity of the yeast ZnT homologue [42], [50]. In the *S. cerevisiae* ZnT2 homologue Zrc1, zinc transport activity was clearly lost by substitution of a leucine to a histidine residue at the position corresponding with one residue before the tryptophan residue at amino acid 152 in hZnT2 [50]. Moreover, the hydrophobic residue at the position corresponding with two residues before the tryptophan in hZnT2 was shown to be involved in dimerization contacts in the X-ray structure of YiiP [42]. These speculations suggest that the W152R hZnT2 mutation may cause interference in the dimerization of hZnT2, and thus result in loss of zinc transport activity.

A number of SNPs have been found in the *SLC30A2/ZnT2* gene, and several of them have been analyzed. Two reported SNPs resulting in L23P and R340C substitutions in hZnT2 may compromise mammary cell functions such as zinc secretion into the milk by changing the subcellular localization of hZnT2, as suggested in the transfection studies [51]. However, their physiological significance regarding the zinc content of breast milk has not been revealed. Moreover, two other SNPs have been reported to be associated with mildly reduced milk zinc (∼10% reduction) in Chinese mothers [52]. One of these was found in the promoter region, causing a –697G>T that may reduce *hZnT2* transcription, but we found no such substitution in the Japanese mother in this study (data not shown). We could not confirm mutations in and around the promoter region and four potential STAT5 binding sites of the *SLC30A2/ZnT2* gene in the mother (See Figure 3 and data not shown). These results strongly exclude the possibility that reduction or dysregulation of h*ZnT2* transcription was the cause of the low milk zinc in our case.

Milk zinc concentrations are considerably higher than those of the maternal serum [53]. Thus, effective mechanisms facilitating the secretion of large amounts of zinc (1–3 mg zinc/day) into the milk operate during lactation in mammary epithelial cells [54], [55]. ZnT2 plays a major role in this process in humans, as described above. Similar extraordinary transport of zinc into secretory vesicles is found in synaptic vesicles in neurons and insulin granules in pancreatic β-cells [56], [57], where zinc has critical physiological functions [55], [58], [59]. In these cells, ZnT3 and ZnT8, which are highly homologous to ZnT2 [60], play critical roles in zinc transport [61], [62]. Both have attracted interest because hZnT3 is proposed to be associated with Alzheimer's disease [63], [64]. In addition, mutation of hZnT8 is involved in both type I and type II diabetes mellitus [65], [66]. The tryptophan residue at amino acid 152 (corresponding to W152 in hZnT2) and the serine residue at amino acid 296 (S296) investigated in this study, and the histidine residue at amino acid 54 (H54) and glycine residue at amino acid 87 (G87) found in previous studies are highly conserved in both hZnT3 and hZnT8 (Figure 2). Particularly, the conservation between hZnT2 and hZnT3 is complete. Thus, substitutions of these residues are likely to cause impairment of the zinc transport function in both hZnT3 and hZnT8, which may be implicated in disease pathogenesis. However, we found no SNPs at those positions in either gene, based on alignment search of the SNP database between ZnT2 and ZnT3 or ZnT8. Information on mutations in the *SLC30A3/ZnT3* or *SLC30A8/ZnT8* genes that may result in functional changes also would be useful for predicting the risk of low zinc content in breast milk. This approach would be helpful in understanding the pathogenesis related with perturbed zinc homeostasis and ZnTs.

Our results mark an important step forward in the understanding of the molecular mechanism behind zinc deficiency in a breast-fed infant. ZnT2 doubtlessly plays a critical role in zinc secretion into milk in humans, but other ZnT transporters may contribute to this function. Significantly decreased h*ZnT5* and h*ZnT6* mRNA was found in fibroblasts and lymphoblasts in two mothers secreting zinc-deficient milk [22]. This suggests that hZnT5-hZnT6 heterodimers contribute to the etiology of neonatal zinc deficiency in different manner than hZnT2. Although direct evidence for the contribution of hZnT4 to the zinc concentration in human breast milk is lacking, its functions in mammary glands are slowly being characterized [67], [68]. Comprehensive understanding of the molecular basis of the relationships between ZnT2 and these ZnT transporters in mammary epithelial cells is required to protect breast-fed infants against zinc deficiency, and to aid in their optimal growth and development.

## Supporting Information

Figure S1 **Affected infant showing erythematous and erosive dermatitis around the diaper region, neck and fingers**.
(TIFF)

Table S1 **Primers used for sequencing of the** ***SLC30A2/ZnT2*** **gene**.
(DOC)

Table S2 **Primers used for sequencing of the** ***SLC30A4/ZnT4*** **gene**.
(DOC)
