# Supplementary figures and images for: Compound Heterozygous Mutations in SLC30A2/ZnT2 Results in Low Milk Zinc Concentrations: A Novel Mechanism for Zinc Deficiency in a Breast-Fed Infant
Source: PLoS One. 2013 May 31;8(5):e64045. doi: 10.1371/journal.pone.0064045 (PMC3669329; doi:10.1371/journal.pone.0064045)

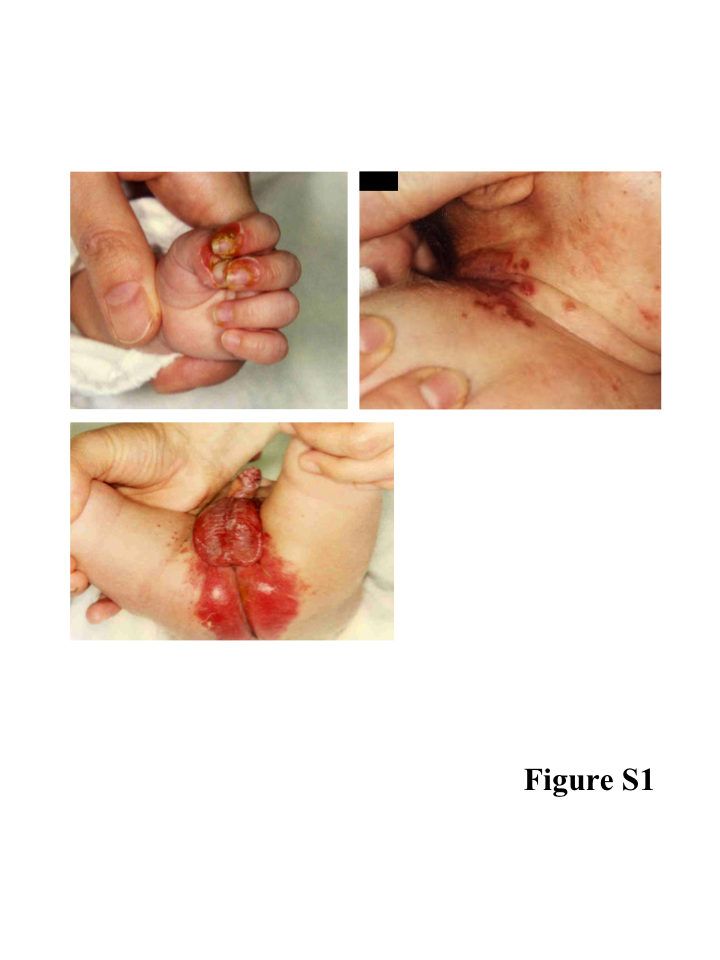

Supplement: Figure S1 — Affected infant showing erythematous and erosive dermatitis around the diaper region, neck and fingers. (TIFF) [file pone.0064045.s001.tiff]
