# Supplementary material for: Compound Heterozygous Mutations in SLC30A2/ZnT2 Results in Low Milk Zinc Concentrations: A Novel Mechanism for Zinc Deficiency in a Breast-Fed Infant
Source: PLoS One. 2013 May 31;8(5):e64045. doi: 10.1371/journal.pone.0064045 (PMC3669329; doi:10.1371/journal.pone.0064045)
Supplement: Table S1 — Primers used for sequencing of the SLC30A2/ZnT2 gene. (DOC) [file pone.0064045.s002.doc]

**Table S1. Primers used for sequencing of the *SLC30A2/ZnT2* gene**

| Exon | primers | Sequence (5’ to 3’) |
| --- | --- | --- |
| 1,2 | ZnT2-ex1-Fw | GAGACACGGGAGCGCTTGGCACGCGGAGCC |
| 1 | ZnT2-ex1-Rv | CTGGGCTGCGCCCCAAGGGAGAGACGGTCC |
| 2 | ZnT2-ex2-Rv | CCATGTGAGAACACAGGTTGTTGTTAGACC |
| 3 | ZnT2-ex3-Fw | GCCTGTGGTCTCCCTGCTGCACACACAGTC |
| 4 | ZnT2-ex4-Fw | GTGGGAGGTGGGTGGGGAGGATCCTGAAGG |
| 3, 4 | ZnT2-ex4-Rv | GCAGACATAGGTGTGGGTGTGAGAGGCGGG |
| 5, 6 | ZnT2-ex5-Fw | GGGGCTTGAGATTTTTGCCCTACAAGTTGG |
| 5, 6 | ZnT2-ex6-Rv | CTGGCTCCCCGCCCATGTGCTAGGATGCCC |
| 7, 8 | ZnT2-ex7-Fw | GACACCTGAGGATCAGGAGCCAGCCCTGCA |
| 7 | ZnT2-ex7-Rv | ACCTGGACCCGTTGGGGATGGCACTAGGCC |
| 8 | ZnT2-ex8-Rv | GTGCCTATTGCTATAGGCAGATGGAGGGGC |
| 8 | ZnT2-ex8-Fw | GGGCCAACTCTGTTGCCTACCTGGCCTGAC |
| 8 | ZnT2-ex8-Rv-II | ACTTGGCCAACTGGCTCTTGTTCTCAACCC |
