# Supplementary material for: Compound Heterozygous Mutations in SLC30A2/ZnT2 Results in Low Milk Zinc Concentrations: A Novel Mechanism for Zinc Deficiency in a Breast-Fed Infant
Source: PLoS One. 2013 May 31;8(5):e64045. doi: 10.1371/journal.pone.0064045 (PMC3669329; doi:10.1371/journal.pone.0064045)
Supplement: Table S2 — Primers used for sequencing of the SLC30A4/ZnT4 gene. (DOC) [file pone.0064045.s003.doc]

**Table S2. Primers used for sequencing of the *SLC30A4/ZnT4* gene**

| Exon | primers | Sequence (5’ to 3’) |
| --- | --- | --- |
| 1,2 | ZnT4-ex1-Fw | GCAGGGCGGGGAGAGGCGGTGGCTGTGGGC |
| 1 | ZnT4-ex1-Rv | GCCGCTGGCGGCGGGTCGCAGGGCCGACCC |
| 2 | ZnT4-ex2-Rv | CGGTAGATGACAGTGGTTGAACAACTAGAA |
| 3 | ZnT4-ex3-Fw | GGCAGAGGTTGCAGTGAGCTGAGATTGCAC |
| 3 | ZnT4-ex3-Rv | TTCTGATGATATCTTCAGGACATAGGGAGC |
| 4, 5 | ZnT4-ex4-Fw | GTTGGTATGAAGTGTAGTAACCATGCTGAA |
| 4, 5 | ZnT4-ex5-Rv | CTGATATGACAATCAGCCTTAGTTTTCATG |
| 6, 7 | ZnT4-ex6-Fw | GGGACATATTCCTTGCTTTGCTTTGCAGTC |
| 6, 7 | ZnT4-ex7-Rv | GCCTTTTGTTTAAGGCTGTTAACAAAAGCC |
| 8 | ZnT4-ex8-Fw | CCCAGTATATTTCAGTTGTTTTTTTCCCTG |
| 8 | ZnT4-ex8-Fw-II | CTCGACACCCAGCTTCTGGAATTGCTGCTT |
| 8 | ZnT4-ex8-Fw-III | CCCACCTTTCACATATAGTTCAACAACATT |
| 8 | ZnT4-ex8-Rv-II | CTCGAGGGATACTTCCACCTTTGCCTCCCC |
| 8 | ZnT4-ex8-Fw-IV | CTGCAGCCTGAGTAACAAAATGAGACCCTA |
| 8 | ZnT4-ex8-Rv | TTTTTTAAGGAACAGGTCGATACAAAAGCG |
